# Self-hybridisation between interband transitions and Mie modes in dielectric nanoparticles

**DOI:** 10.1515/nanoph-2023-0781

**Published:** 2024-02-01

**Authors:** Christos Tserkezis, P. Elli Stamatopoulou, Christian Wolff, N. Asger Mortensen

**Affiliations:** POLIMA–Center for Polariton-driven Light–Matter Interactions, University of Southern Denmark, Odense M, Denmark; D-IAS–Danish Institute for Advanced Study, University of Southern Denmark, Odense M, Denmark

**Keywords:** self-hybridised polaritons, Mie resonances, interband transitions

## Abstract

We discuss the possibility of self-hybridisation in high-index dielectric nanoparticles, where Mie modes of electric or magnetic type can couple to the interband transitions of the material, leading to spectral anticrossings. Starting with an idealised system described by moderately high constant permittivity with a narrow Lorentzian, in which self-hybridisation is visible for both plane-wave and electron-beam excitation, we embark on a quest for realistic systems where this effect should be visible. We explore a variety of spherical particles made of traditional semiconductors such as Si, GaAs, and GaP. With the effect hardly discernible, we identify two major causes hindering observation of self-hybridisation: the very broad spectral fingerprints of interband transitions in most candidate materials, and the significant overlap between electric and magnetic Mie modes in nanospheres. We thus depart from the spherical shape, and show that interband–Mie hybridisation is indeed feasible in the example of GaAs cylinders, even with a simple plane-wave source. This so-far unreported kind of polariton has to be considered when interpreting experimental spectra of Mie-resonant nanoparticles and assigning modal characters to specific features. On the other hand, it has the potential to be useful for the characterisation of the optical properties of dielectric materials, through control of the hybridisation strength via nanoparticle size and shape, and for applications that exploit Mie resonances in metamaterials, highly-directional antennas, or photovoltaics.

## 1 Introduction

Polaritonics is currently one of the most rapidly growing areas in nanophotonics [1], [2], but also one of the richest in physics [3], [4]. With strong light–matter interactions lying at its heart, polaritonics has drawn inspiration from quantum optics [5] to evolve into an intriguing and fruitful combination of electrodynamics, condensed-matter physics, and chemistry. Over the years, a plethora of strong-coupling configurations has been introduced, ranging from semiconductor quantum wells [6], [7] and quantum dots [8], [9] coupled to mirror or Bragg-reflector cavities, to excitons in organic molecules [10], [11], [12] and two-dimensional (2D) materials [13]–[16] interacting with photonic crystals or plasmonic nanostructures. The exciting possibilities that open by the interplay between some material mode and confined light, and the subsequent formation of half-light–half-matter states, is widely explored for applications related to quantum and neuromorphic computing [17], [18], molecular photophysics [19] and polaritonic chemistry [20], [21], control of optical chirality [22], [23], Bose–Einstein condensation [24], [25] and polariton lasing [26], [27].

Excitons are not the only matter components that can produce polaritons. In principle, every polar excitation in a material can couple strongly to the optical modes of a closed or even open cavity. Among the most characteristic examples are phonon polaritons [28], where phonons in the bulk or in van der Waals materials [29], [30] can couple either directly to light or to excitons in molecules [31]. Similarly, spins in microwave cavities produce magnons [32], the collective excitation of electrons in different subbands leads to intersubband polaritons [33], and Landau-level transitions in 2D electron gases can couple to electric resonators and, under the influence of a strong magnetic field, produce Landau polaritons [34]. More complex polaritons involving the coupling of two quasiparticles (one of the two usually being an exciton) include plexcitons in noble metal nanoparticles (NPs) covered with an excitonic layer [35], perovskite-exciton polaritons [36], and Mie-excitons in high-index dielectric NPs [37], [38], possibly with a magneto-optical character [39].

Recently, it was shown that an optical cavity is not strictly required to achieve the modal coupling that produces polaritonic states [40]. Indeed, different optical modes in the same system can couple to each other, just upon external excitation, e.g. with a laser [41]. Such modes can be a combination of electronic or photonic with vibrational modes [42], but self-hybridisation can also occur between Mie [43] and vibrational modes in a water droplet [44]. A similar effect in large dielectric spheres is related to excitons in semiconductors interacting with the whispering-gallery modes of the NP [45], or directly with Mie modes in transition-metal-dichalcogenide (TMD) nanodiscs [46]. Furthermore, it can be observed in the coupling of Fabry–Pérot resonances of TMD [47], [48] or perovskite [49] flakes to their excitonic modes. Inspired from these works, strong coupling was recently observed between localised surface plasmons (LSPs) and interband transitions (IBTs) in nickel (Ni) films and NPs [50], [51]. In view of these latest endeavours, it is reasonable to wonder why self-coupled polaritons have never been reported in high-index dielectrics such as Si, where spherical or cylindrical NPs support a richness of optical modes of both electric and magnetic character [52], [53], and have been shown to be more flexible that plasmonic NPs in terms of light–matter coupling [54].

In this work, we show that interband–Mie coupling is indeed feasible, and explain why its observation is experimentally challenging. We begin with an ideal constant-permittivity sphere, where a superimposed narrow Lorentzian mimics IBT resonances, and examine its optical response with both plane-wave and electron-beam excitation. Having shown, in this case, an anticrossing in both extinction and cathodoluminescence (CL) photon-emission probability, and having thus a clear fingerprint of strong coupling, we shift our attention to the workhorse of Mie-resonant photonics, i.e. silicon (Si) nanospheres. We identify two mechanisms that hinder experimental observation in this case: the very large linewidth of IBT-related resonances in the permittivity of bulk Si, and the fact that electric and magnetic resonances in spherical NPs have significant overlap. To overcome the first issue, we explore other high-index semiconductors like gallium arsenide (GaAs) and gallium phosphide (GaP). For the latter problem, we break the spherical symmetry by designing dielectric pillars, in which magnetic and electric modes can be well separated. Following these actions, we finally manage to successfully identify the self-hybridisation between IBTs and Mie modes. The feasibility of this coupling introduces a new way for analysing the intrinsic material response of nanostructured materials. At the same time, it calls for additional attention when explaining the origin of spectral features and assigning modal characters to them, similarly to the emergence of transition-radiation signals in CL that we reported recently [55].

## 2 Ideal IBT–Mie hybridisation

For a proof of concept, let us begin by considering an ideal situation, of a nanosphere of radius *R* with a constant, moderately high relative permittivity *ɛ* equal to 12. Such an NP resembles Si in the infrared part of the spectrum [52], but more interesting physics takes place in the visible and ultraviolet (UV) [56], including IBTs that result in a negative permittivity in the UV and effectively lead to a plasmonic behavior [57]. To mimic this, we add to *ɛ* a narrow Lorentzian term, so that the total, dispersive permittivity becomes

(1) $$\varepsilon \left(\omega \right)=\varepsilon_{\infty }-\frac{f\omega_{c}^{2}}{\omega^{2}-\omega_{c}^{2}+i\omega \gamma_{c}}.$$

**Figure 1:**
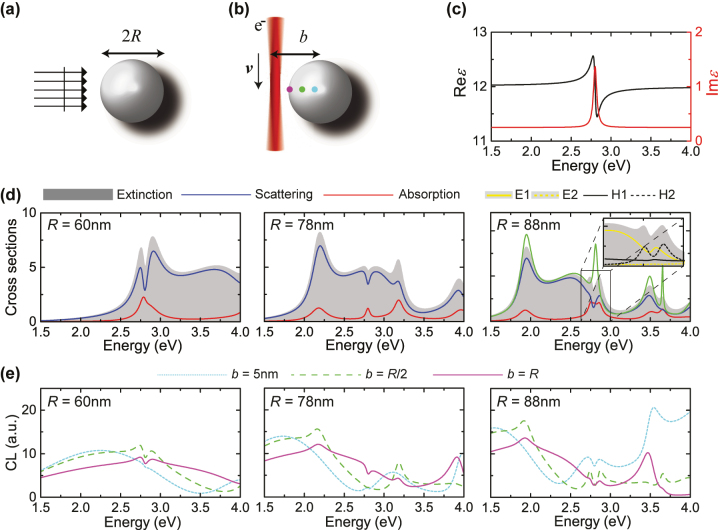
Mie-IBT hybridisation in an ideal system. (a) A spherical NP of radius *R* excited by a plane wave. (b) The same NP as in (a), excited by an electron beam with velocity *v* = 0.33*c*, passing near or through the NP at impact parameter *b*. The coloured dots denote the three excitation spots corresponding to the spectra shown in (e). (c) Real (black line, left vertical axis) and imaginary (red line, right axis) part of the permittivity of Eq. (1). (d) Normalised (to the geometrical cross section *πR* ^2^) extinction (grey shaded areas), scattering (blue lines), and absorption (red lines) cross sections, for spherical NPs described by the permittivity of (c), for three different radii *R* = 60 nm (left panel), *R* = 78 nm (middle panel), and *R* = 88 nm (right panel). In the rightmost panel, the green line denotes the spectrum of an NP with the same radius but in the absence of IBTs (*ɛ* = *ɛ*_∞_ = 12). The inset zooms into the frequency window of the magnetic quadrupolar mode (H2), and decomposes the extinction spectrum into contributions from electric (E1 and E2) and magnetic (H1 and H2) dipoles and quadrupoles (yellow solid and dashed, and black solid and dashed lines, respectively). (e) CL spectra (arbitrary units) for the same NPs as in (d), for three different impact parameters: grazing (*b* = *R*, purple solid lines), traversing at *b* = *R*/2 (green dashed lines), or traversing near the center (*b* = 5 nm, light-blue dotted lines) of the NP.

In the above, *ω* is the angular frequency of the incident light, *f* = 0.02 is the oscillator strength in the Lorentz model, *ω*_c_ is the resonance frequency, *γ*_c_ the damping rate, and *ɛ*_∞_ = 12. In order to have a reasonably broad resonance in the frequency region where the first few Mie modes of NPs with radii varying from 50 to 100 nm appear, yet without disrupting the constant-*ɛ* picture much, we choose *ℏω*_c_ = 2.8 eV, and *ℏγ*_c_ = 0.05 eV. The real and imaginary part of *ɛ* described in Eq. (1) are plotted in Figure 1c, corresponding indeed to a nearly constant permittivity with a narrow resonance at 2.8 eV.

The nanosphere can be excited by either an incident plane wave, as sketched in Figure 1a, or by a swift electron beam, passing near or through the NP with impact parameter *b*, as shown in Figure 1b. In what follows, we assume that the electron beam is modelled as a single electron travelling with speed *v* = 0.33*c* (with *c* being the speed of light in vacuum); such a velocity corresponds to a low acceleration voltage of 30 kV, which is typical in CL experiments. The photon-emission probability for an electron emitted by the NP once it has been excited by the electron beam is calculated here semi-analytically, with the Mie-based method described in Ref. [58].

In Figure 1d we scan over different NP radii, so as to gradually match each of the first few Mie modes – excited here with a plane wave – to the energy of the Lorentzian. The first such mode is of magnetic dipolar character (H1), originating from the emergence of displacement currents inside the NP due to the excitation of bound charges in its bulk, with a phase difference as a result of retardation [53]. For a radius *R* = 60 nm (left-hand panel in Figure 1d), the H1 mode indeed has its resonance at 2.8 eV; as a result of its interaction with IBTs, a structure of two resonances with a dip in between them appears in the extinction cross section spectrum (grey shaded area). A similar anticrossing is present in the scattering cross section (blue line), which, due to the nearly negligible losses in the bulk of the NP, dominates the extinction spectrum. At the same time, the absorption cross section is characterised by a broad resonance peaking at 2.78 eV, with a shoulder at 2.88 eV. This feature in absorption suggests that indeed, there is interaction between the two modes, albeit relatively weak; in principle, it would be stronger if the NP were more lossy [37].

Subsequently, in the middle panel of Figure 1d we increase the radius to *R* = 78 nm, bringing the centre of the broad electric dipolar (E1) Mie mode to the frequency of interest. What we see is a small dip in extinction and scattering, accompanied by just a single resonance in absorption, practically of the same absolute value as the “hole” left in the scattering spectrum. Clearly, there is no hybridisation between the two modes in this case, which is typical of what has been termed enhanced or induced absorption [59]. We have not brought the two modes to interact, but rather we have added one mechanism to subtract some of the energy corresponding to E1.

Finally, we increase the radius even further, to *R* = 88 nm, for which it is the magnetic quadrupolar mode (H2) of the NP that resonates at 2.8 eV. In this case, the fingerprint of hybridisation is much better visible, since there is a double-peaked resonant feature in both scattering and absorption spectra. In the right-hand panel of Figure 1d, we also include, with a green line, the extinction spectra in the absence of any IBTs, i.e. when we set *f* = 0 in Eq. (1), to better demonstrate the two uncoupled systems (H2 resonance in green line in Figure 1d, and IBT resonance in Figure 1c). As further proof that it is indeed the same mode, H2, that has split in two due to its interaction with the IBTs, the inset of the panel presents a decomposition of the extinction spectrum into multipolar contributions, E1, E2, H1, and H2 (E2 being the electric quadrupole). The E1 mode exhibits again the tail of enhanced absorption discussed above (yellow solid line), but H2 (black dashed line) is perfectly split into two hybrid resonances, shifted by 0.05 eV on either side of the material resonance.

Since IBTs describe a loss mechanism directly related to processes inside the bulk of the material, it is reasonable to ask whether the hybridisation discussed above would be easier to observe with a localised excitation, such as a focused electron beam passing at a grazing trajectory, or even better penetrating inside the NP. In the three panels of Figure 1e we repeat the same radius-dependence study as in Figure 1d, but this time for CL calculations, for three different impact parameters *b*: a grazing one (purple lines), a beam travelling inside the NP at distance *b* = *R*/2 from the center (green lines), and one crossing the NP nearly at its centre (light-blue lines) – we set *b* = 5 nm instead of *b* = 0 for numerical reasons, to ensure converged spectra. What we see in this case is that for both the *R* = 60 nm and the *R* = 78 nm NP, relatively narrow dips appear at 2.8 eV, for both the *b* = *R*/2 and the *b* = *R* electron trajectories. But these are quite narrow to attribute to a hybridisation; the reason for this weak interaction is rooted to the broadness of the spectral features in CL, which is made worse for penetrating trajectories due to the interference with transition radiation [55]. This is most clearly visible in the case of the *b* ≃ 0 trajectory, where essentially only this periodic oscillation is visible. Nevertheless, for the largest of the three NPs, *R* = 88 nm in the right-hand panel of Figure 1e, it can still be seen that the H2 mode hybridises with IBTs, and the strength of the interaction becomes larger for the penetrating trajectory *b* = *R*/2. This suggests that CL spectroscopy could be used as an alternative to plane-wave excitation in what follows, where we shall explore realistic materials to establish if and when one should anticipate observing this behaviour in experiments. Nevertheless, one should also keep in mind that, in our calculations, the electron beam is modelled as a single electron travelling along a straight line, free of any collisions. In realistic materials, one should always consider the possibility of the electron beam transferring momentum and thus inducing indirect IBTs.

## 3 Spherical particles of different materials

### 3.1 Silicon

**Figure 2:**
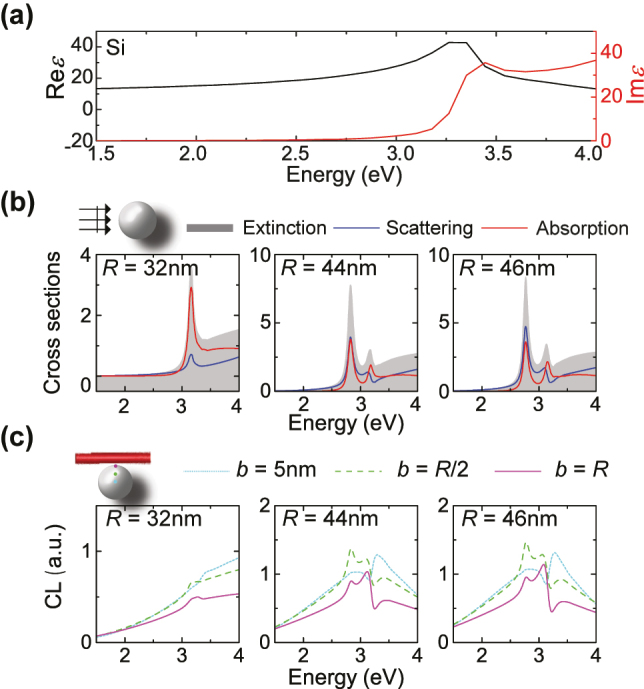
Absence of Mie-IBT hybridisation in Si nanospheres. (a) Real (black line) and imaginary (red line) part of the relative permittivity of bulk Si [56]. (b) Normalised extinction (grey shaded areas), scattering (blue lines), and absorption (red lines) cross section of Si nanospheres of radii *R* = 32, 44, and 46 nm (left, middle, and right panel, respectively), upon plane-wave excitation. (c) CL spectra (arbitrary units) for the same NPs as in (b), for three different impact parameters: grazing (*b* = *R*, purple solid lines), traversing at *b* = *R*/2 (green dashed lines), or traversing near the centre (*b* = 5 nm, light-blue dotted lines) of the NP.

Si is the dominant material in all theoretical and experimental activities regarding Mie-resonant systems [60], [61], [62], owing mostly to its availability in nature and its extensive use in modern electronics. High-quality Si nanospheres with limited surface roughness and good control over their dimensions are nowadays experimentally feasible [63], enabling the detailed and accurate characterisation of their optical properties. Nevertheless, IBT–Mie resonance coupling has never been reported. The reason can be easily understood by observing the permittivity of bulk silicon, shown in Figure 2a (reproducing data from Ref. [56]). The onset of IBTs appears at around 3.2 eV, but it is characterised by a very broad, blunt resonance, and considerable losses (captured by the imaginary part of the permittivity) all the way to the deep UV. This already suggests that any geometrical resonance (such as the Mie modes of interest here) will be severely damped and only weakly couple to the IBT resonance.

To make the situation even worse, the fact that IBTs appear at such high energy means that small Si NPs are required in order to align any Mie modes with them. This is indeed shown in the far-field spectra of Figure 2b, where we show that radii as small as ≈30 nm are needed for the H1 mode to match the energy of IBTs, and already at *R* ≤ 50 nm the – hardly distinguishable from E1 – H2 mode has also redshifted farther than this energy. But for such small NPs, Mie resonances (particularly the magnetic ones) are very weak: as we discussed above, retardation plays a key role in the creation of the displacement current which triggers these resonances, and radii above 50 − 60 nm are desirable, as is also shown in Figure 1. It is then no real surprise that no hybridisation can be observed in far-field spectra, for none of the H1, E1, H2 modes (left, middle, and right panel of Figure 2b). But the same can be said for electron-beam excitation and the corresponding CL spectra, shown in Figure 2c, again for three characteristic electron-beam trajectories. Even though penetrating electron trajectories could potentially excite the modes more efficiently (as seems to be the case for the *b* = *R*/2 impact parameter, green spectra in the figure), no evidence of modal interaction can be observed – the sharp dip at about 3.2 eV is related to the Kerker condition [64] and the interference between the dipolar Mie modes of the NP.

### 3.2 Other high-index semiconductors

**Figure 3:**
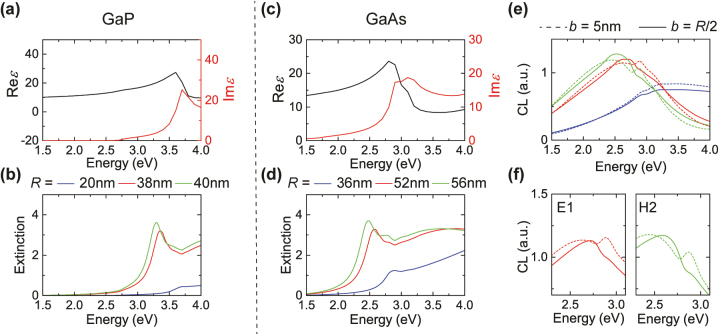
Exploration of other semiconductors: GaP and GaAs. (a) Real (black line) and imaginary (red line) part of the relative permittivity of bulk GaP [65]. (b) Normalised extinction cross section of GaP nanospheres of radii *R* = 20, 38, and 40 nm (blue, green, and red lines, respectively). (c) Real (black line) and imaginary (red line) part of the relative permittivity of bulk GaAs [65]. (d) Normalised extinction cross section of GaAs nanospheres of radii *R* = 36, 52, and 56 nm (blue, green, and red lines, respectively). (e) CL spectra (arbitrary units) for the same NPs as in (d) (same colour coding), for two different impact parameters: traversing at *b* = *R*/2 (solid lines), or traversing near the centre (*b* = 5 nm, dashed lines) of the NP. (f) Contribution to the spectra of the *R* = 52 nm NP of panel (e) from the electric-dipolar (E1) (left-hand panel) and the magnetic-quadrupolar (H2) (right-hand panel) mode only.

Since we attributed the absence of any IBT–Mie hybridisation in Si to the intrinsic properties of the material and, in particular, the high (for our needs) energy at which IBTs appear and the large linewidth of the resonance (if any resonance can be identified above 3.5 eV), it is reasonable to explore other high-index materials. To this end, we resort to Ref. [65], which is devoted to the measurement and characterisation of most traditional semiconductors. Out of the materials encountered there, we present in Figure 3 two cases: one that is not satisfactory for our goal (GaP), and one that suggests that the desired hybridisation could be visible (GaAs). Among the other possibilities, germanium (Ge) has a resonance around 2.1 eV, albeit too broad for our needs, while indium (In)-based compounds are characterised by more than one well-defined resonances in a narrow energy window, and would not allow for a clear interpretation of the calculated spectra. The GaP example, depicted in Figure 3, exhibits a relatively blunt resonance around 3.5 eV, as can be seen in the permittivity of Figure 3a. Both of these characteristics make it a rather bad choice for our study and, indeed, the extinction spectra of Figure 3b do not suggest any hybridisation with the (rather weak for these radii) Mie modes.

Unlike the aforementioned materials, GaAs appears to be a more appropriate candidate in our quest. Its permittivity is dominated by a relatively sharp resonance at lower energy, around 2.7 eV, while a second resonance at slightly higher energy only appears as a shoulder in the real part (Figure 3c). The extinction spectra of NPs with appropriate (larger than in the case of GaP) radii (*R* = 36 nm – blue line, *R* = 52 nm – red line, and *R* = 56 nm – green line) suggest that there might be a possibility of interaction of all three modes, especially E1 and H2, although it is not yet entirely clear whether the resonance splitting indeed occurs, or it is mostly wishful thinking. To further explore the situation, we calculate CL spectra for two different penetrating electron-beam trajectories (*b* = *R*/2 – solid lines, and *b* = 5 nm – dashed lines), as shown in Figure 3e for all three NP sizes. Resonance splitting is now more visible for the E1 and H2 modes, particularly when the electron beam travels near the center of the NP (in which case, the H1 mode is very weakly excited). The two panels of Figure 3f zoom into these two splittings, showing only the contribution to the CL spectrum from the corresponding term in the multipolar expansion of the field, suggesting that it is indeed every individual mode coupling to the IBTs. Nevertheless, this is still a rather weak effect, which could be masked in any experiment by fabrication imperfections and material damping. What makes the interpretation of the spectra particularly challenging, is the significant overlap between different multipoles. To eliminate this source of ambiguity, we depart in what follows from the spherical NP shape, and design cylindrical pillars with a moderate aspect ratio, known to display well-separated magnetic and electric Mie modes [66].

## 4 Separation of multipoles in cylinders

**Figure 4:**
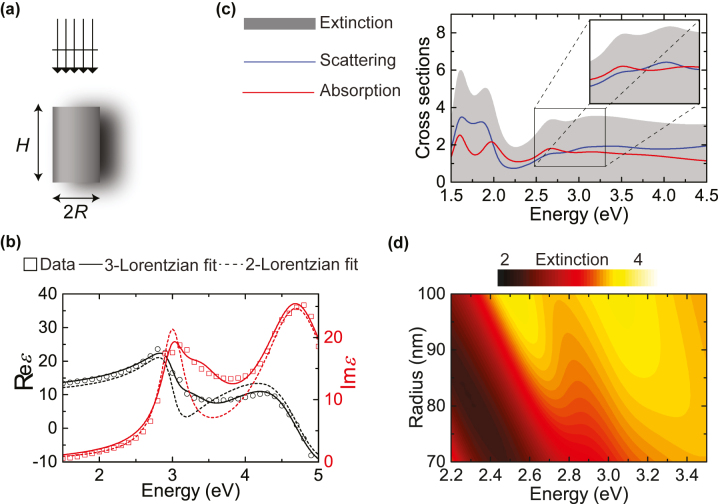
Mie-IBT hybridisation in GaAs cylinders. (a) Schematic of a GaAs cylindrical pillar with radius *R* and height *H*, illuminated by a plane wave coming from top. (b) Real (black open circles) and imaginary (red open squares) part of the permittivity of GaAs as obtained from experiment [65], and Lorentzian fits to the data, with 3 (solid lines) or 2 (dashed lines) Lorentzian oscillators. (c) Extinction (grey shaded area), scattering (blue line) and absorption (red line) cross section (normalised to the geometric cross section of a sphere with the same volume) for a GaAs pillar as the one sketched in (a), for *H* = 200 nm and *R* = 84 nm, described by the permittivity given by solid lines in panel (b). The inset zooms in the energy window where the E1 mode interacts with IBTs in GaAs, which is now described by the permittivity shown with dashed lines in panel (b). (d) Contour map of extinction of GaAs pillars with *H* = 200 nm, as a function of radius and energy, in the frequency region of IBTs.

In Figure 4 we move from a spherical to a cylindrical shape. We design cylindrical pillars of radius *R* and height *H*, illuminated with a plane wave from the top, as shown in the schematics of panel (a). We focus on GaAs, as the most promising candidate according to the discussion in the previous section. Instead of directly interpolating the experimental data shown in Figure 3c, we choose to fit those data with a multi-Lorentzian permittivity, similar to that of Eq. (1), with *ɛ*_∞_ = 2.169 and three oscillator terms with *f*_1_ = 1.490, *f*_2_ = 2.379, and *f*_3_ = 6.002, resonant at *ℏω*_1_ = 3.007 eV, *ℏω*_2_ = 3.398 eV, and *ℏω*_3_ = 4.731 eV; the corresponding damping rates are *ℏγ*_1_ = 0.399 eV, *ℏγ*_2_ = 0.836 eV, and *ℏγ*_2_ = 1.166 eV. As can be seen in Figure 4b, the fitting (solid lines) to the experimental data is very good, and should reproduce accurately all NP resonances. The reason for fitting the data is that it conveniently allows us to selectively deactivate some of the features at will. In particular, as we discuss next, it is useful to be able to disregard oscillator 2, and the corresponding resonance expected at about *ℏω*_2_ = 3.398 eV; the corresponding permittivity is shown in Figure 4b with dashed lines, where, in addition, we had to increase *f*_1_ to 2.5 to compensate for the removal of the strong background that oscillator 2 adds to the resonance of oscillator 1. This choice, which reproduces very well the experimental data for energies below 3.4 eV, will allow in what follows an unambiguous interpretation of all spectral features. For the calculation of the spectra, traditional Mie theory is obviously no longer applicable; we thus employ the nearest equivalent, namely the extended boundary condition method (EBCM) [67], which is still based on spherical-wave expansions. The simulation set-up and all convergence parameters are as described in Ref. [38]. We should note here that, within EBCM, the characterisation of modes in terms of multipoles is straightforward, because the matrix that connects the scattered to the incident field, though not diagonal, is almost always dominated by a single element with specific angular-momentum indices *ℓ* and *m*; alternatively, one can use Cartesian multipoles [68].

In Figure 4c we plot the extinction (grey shaded area), scattering (blue line), and absorption (red line) spectra of a pillar with *R* = 84 nm and *H* = 200 nm, a height that, according to our calculations, is long enough to adequately separate the different dipolar modes. Indeed, the E1 mode appears now at about 2.8–3.0 eV, while H1 appears at much lower energy, between 1.5 and 2.0 eV; the cylindrical shape has in fact lifted the degeneracy of the mode in terms of magnetic angular momentum *m* number, and we now observe two separate resonances. But, more importantly, the E1 mode seems to indeed interact with IBTs at about 3.0 eV, and it produces a resonance splitting, in both scattering and absorption, suggesting a real hybridisation. Exploiting the spherical-wave decomposition that is intrinsic in EBCM, we can confirm that the two resonances come from the same *ℓ* and *m* angular–momentum elements. Another factor that need be considered is the possibility of the two resonances being related to the two different resonances in the permittivity of GaAs, at frequencies *ω*_1_ and *ω*_2_. This is where our fitting to the experimental data proves important, since we can just turn the resonance at *ω*_2_ off (by setting *f*_2_ = 0), and explore the same system anew. The corresponding spectra, just in the energy window of interest, are shown in the inset of Figure 4c, and indeed show the same double-resonant behaviour. Finally, Figure 4d shows a contour plot of extinction versus nanocylinder radius (always for the same height), and how the two hybrid resonances emerge as we change the radius and tune the E1 mode across the energy of IBTs.

It appears, therefore, that observation of hybridisation between Mie modes and IBTs should be possible, at least in select conventional materials, whose IBTs lie at energies for which Mie resonances have fully developed, and in NP geometries that reduce the overlap of these resonances. Such observations should be feasible both in scattering and absorption measurements, but also in electron-beam spectroscopies, since the crystal momentum is typically a few orders of magnitude smaller that of the electrons [69], and only direct transitions would be enabled. On the other hand, the emergence of this hybridisation could be exploited in the characterisation of novel materials, where resonance splitting, varying with NP size and shape, could provide information about the strength and linewidth of resonances related to IBTs. Apart from this potential, the emergence of two hybrid absorption bands close in energy could be exploited in photovoltaics [70], [71]. Similarly, many of the applications in which Mie-resonant NPs are used as antennas depend on the Kerker effect, and the capability to control the directionality of scattered light with destructive or constructive interference of different multipoles [64], [72]; if those modes further interact with IBTs, completely new interference conditions are anticipated to emerge. Finally, one cannot exclude the possibility of Mie–IBT hybridisation affecting all-dielectric metasurface-based colouring [73]. Nevertheless, all these options and possible applications remain at the moment within the realm of hypothesis; for the time being, experimental verification, and possibly material engineering to better demonstrate the effect, are the first steps to be considered.

## 5 Conclusions

In summary, inspired from recent activities in self-hybridised polaritons, we posed the question why no hybridisation between Mie modes in high-index dielectric NPs and their intrinsic IBTs has ever been reported. By considering an ideal, lossless, constant-*ɛ* sphere, we realised that this hybridisation should indeed be feasible. We identified two major factors that hinder its experimental observation: the very broad resonances that characterise IBTs in most traditional high-index dielectrics, and the significant overlap of Mie resonances in spherical NPs. To deal with the former, we considered a large variety of semiconductors, and identified GaAs as the most suitable candidate. For the latter, we departed from the spherical shape, and showed a clear hybridisation in GaAs cylindrical NPs, with the effect in question being better observable in the interaction of the E1 mode with IBTs. Recognition of this possibility introduces another element of which one should be aware when characterising NPs in terms of their modes.
